# Medical Jurisprudence and Toxicology

**Published:** 1843-01

**Authors:** 


### MEDICAL JURISPRUDENCE AND TOXICOLOGY.

*On the Recent Discoveries Relative to the Chemical Processes for detecting Arsenic.*
By A. Devergie.

Among the numerous processes lately examined by the committee of the Academy of Sciences, appointed to investigate the subject of arsenical poisoning, one suggested by M. Devergie in 1840, in his treatise on Legal Medicine, was omitted. As the committee reported their approval of one process in which a large quantity of sulphuric acid is necessarily employed, it is right (observes M. Devergie,) to place before them an account of those researches made by me, to show that the use of this acid is decidedly objectionable in all such cases. In examining the viscera of Nicholas Mercier in 1839, arsenic was discovered both by the use of nitrate of potash and sulphuric acid. The nitrate of potash was then tried with sulphuric acid alone, and still deposits of arsenic were procured. This was also the case when the purest nitrate of potash which had been expressly prepared by acting on pure potash, with pure nitric acid was used. This led to the suspicion that the source of error was in the sulphuric acid, and that the arsenic was introduced into it in the course of manufacture.

The following is an outline of the process which I have proposed.

1. The animal matter is slowly dried and weighed.

2. It is then to be dissolved by the aid of heat in a solution of pure potash. The arsenious acid, contained in the animal matter, is thereby transformed into fixed arsenite of potash.

3. Nitrate of lime is now to be added to the liquid mass, in the proportion of about two thirds of the weight of the dried animal matter; from this, arsenite of lime, hydrate of lime, and nitrate of potash result.

4, The mixture is evaporated to dryness, and then strongly heated in one or two points ; combustion ensues and slowly spreads throughout the mass. On cooling, the dry residue is treated with muriatic acid until all effervescence has ceased ; water is added, and the solution filtered.

In a well-conducted operation, a colourless liquid easily admitting of filtration is thereby procured. This liquid is introduced into Marsh's apparatus, and should there not be a sufficient evolution of hydrogen, more muriatic acid is added. The first portions of gas will be found to contain hydrogen.

Such is an outline of the process which it was my intention to propose to the Academy of Sciences. *Annales de Hygiène. Jan.* 1842.

[Remarks. The remainder of this paper is purely controversial, and presents no point of interest to the medico-legal student. It is much to be regretted that MM. Orfila and Devergie cannot come to an amicable understanding on the relative merits of their processes for detecting arsenic, more especially since there are strong objections in our views to the methods adopted by both. We have extracted this account of M. Devergie's process, in order to complete the history of the subject, and in justice to that gentleman, to let him speak for himself. In our opinion, the process is too complex to render its adoption advisable in most cases of arsenical poisoning ; and it has the disadvantage of introducing too many foreign substances into the mixture requiring analysis. We do not see that any particular benefit is derived from transforming arsenious acid into arsenite of lime ; or in what respect, this is superior to the original process of Rapp, since practised by Orfila, of deflagrating the dried animal substance with nitrate of potash. In short those who have tried the deflagrating method must have found that a very large quantity of saline matter results, from which it is difficult to free the liquid, without losing arsenic. If the arsenic be in moderately large quantity, and the arsenical matter not very abundant, there is no necessity for a medical jurist to adopt any of these delicate processes for the detection and insulation of the poison ; but where the conditions are reversed, the arsenic being in small, and the animal matter in large proportion ; then although this is the only case in which they would be employed, they are liable to defeat the object of the analyst by overloading the solution with saline matter. For this reason, we object to the plan of M. Devergie, as much as to that proposed by Orfila, and consider that better results may be obtained by resorting to the carbonising process of MM. Dauger and Flaudin. This has already been fully described in a former number of the Review.

---

### *Cases of Asphyxia from Coal Gas.*

By G. Tourdes, Prof. of Legal Medicine at Strasburg.

[There are but very few accurate reports of cases of suffocation from coal gas :—the following will therefore be found interesting to the medical jurist.]

In January, 1841, a whole family, residing at Strasburg, were subjected for forty successive hours to the deleterious action of coal-gas. Five persons, the father, three children, and a servant of the family, died. The mother alone recovered, after a long and severe illness. On closely examining the premises, it was found that the gas had escaped from a pipe which passed near a cellar, immediately below the lodging, in which these people lived. On the discovery of the accident three of the persons appeared to have been dead for some hours, namely, two of the children and a servant. The body of the fourth, a little girl, about five years of age, presented still some traces of warmth. The two others, the father and mother, still breathed; but the father died in twenty-four hours, notwithstanding the most energetic treatment; the mother slowly recovered.

A post-mortem examination of the bodies was made, and there was not much difference in the appearances in the five subjects. The brain and its membranes were congested. The vessels of the pia mater were especially gorged with blood, and the surface of the whole brain was intensely red. The veins of the spinal canal were also congested. In three of the cases, there was considerable effusion of coagulated blood, between the dura mater and the bony canal. The medulla spinalis presented no particular appearance.

The air-passages were strongly injected from the base of the tongue downwards, to the extreme bronchial ramifications. The velum palati and epiglottis were likewise much injected. A thick viscid froth with streaks of blood was found diffused over these parts, except in the case of the father, who had survived twenty-four hours, and had been bled several times. There was one peculiar appearance met with in the whole of these cases. The parenchyma of the lungs was throughout its whole extent of a bright red colour, contrasting strongly with the dark colour of the surface of the organs. The blood was found coagulated in the heart and large vessels, especially in the right auricle. The liver was gorged with blood, and of a deep red colour; in the case of the father, it was pale and of a gray yellow colour. In four of the subjects, the bladder was distended with an enormous quantity of limpid urine. There was nothing remarkable to be seen in the intestinal canal.

Thus then, it will be observed the appearances common to these cases were, coagulation of the blood,—its deep colour,—the bright red colour of the substance of the lungs,—coagulation of the vessels of the brain and of the vertebral sinuses,—a redness of the base of the tongue and fauces, with an abundant frothy mucus streaked with blood in the aerial passages.

As a summary of the effects of coal-gas on the human subject, derived from these and other observations, the following points may be enumerated: vertigo, cěphalalgia, nausea with vomiting, disturbed intellectual faculties with loss of consciousness, general weakness and depression, partial paralysis, convulsions and the usual phenomena of asphyxia slowly appearing but becoming confirmed as life approaches to its termination.

The following is the composition of the Strasburg coal-gas, which led to these fatal accidents:

| | |
|---|---|
| Hydrogen - - - - | 31 |
| Proto-carburetted hydrogen - - - | 22·5 |
| Carbonic oxide - - - - | 21·9 |
| Nitrogen - - - - - | 14· |
| Bicarb. hydrogen and pyrelain - - | 6· |
| Carbonic acid - - - - | 4·6 |
| | 100· |

The preparations of these bodies, although not always the same, do not vary much from the above; for any great variation would materially affect the illuminating power of the gas.

Coal-gas owes its peculiar odour to the pyrogenous carburets of hydrogen, which it contains. It was found by experiment that this odour was well marked, when the proportion of the gas in air amounted only to 1-150th, that it was very perceptible even up to 1-700th, and when the gas formed only 1-1000th part of the mixture by volume, there was a decided impression on the sense of smelling, although this was of a doubtful character.

It might at first view be supposed, that the explosibility of the gas would rather interfere with its asphyxiating properties; but on the occasion of this accident, it was proved that in the apartment in which the individuals were, a stove had been for a long time in active combustion, and a candle had been completely burnt out without producing an explosion in an atmosphere of the gas thus shown to be capable of causing death. Indeed, experiment has established that explosion is liable to take place, only when the gas forms about an eleventh part of the atmosphere.

Experiments were then made on animals to determine the relatively poisonous effects of this gas. In a pure state, coal-gas was found to destroy life immediately, in a proportion of one eighth, it killed a rabbit in five minutes, and a dog in twelve. In a dose of one fifteenth, (a non-explosive proportion,) it killed rabbits in nine, twelve, and fourteen minutes; and pigeons in five minutes. In a proportion of one thirtieth, its action was very evident; but it destroyed life slowly. At one fiftieth, its power was much diminished; and at one seventy-fifth it but slightly affected rabbits; in this last proportion, it was not found to destroy life; and if the experiment be prolonged, we have merely the effects of a vitiated or unrenewed atmosphere.

The chief symptoms in animals, were convulsions, followed by collapse, difficult respiration and drowsiness. On examining the bodies, the lungs, liver, brain, and other organs presented nothing remarkable; but firm coagula of blood, were commonly found in the heart.

An attempt was then made to determine to which of the components of coal-gas, its fatal action on the economy was due. After many experiments, M. Tourdes inferred that the deleterious agent was the carbonic oxide.

There can be no doubt, that coal-gas has a directly poisonous action, and that it is not a mere asphyxiating agent, like hydrogen or nitrogen. Thus it begins to affect the system, even when present in very small proportion; and the experiments already related, certainly show that it could not be substituted for nitrogen in air, without destroying life very speedily; therefore the irresistible inference is, that it is a *direct* poison. The experiments performed on animals as well as the chemical analysis appear to prove, that the carbonic oxide is the principal cause of the energetic action of coal gas. This is the agent which affects the system most powerfully and rapidly. The pyrelains may contribute to its operation, but not to any well-marked degree; the bicarburetted hydrogen has but little influence, the proto-carburetted hydrogen still less, while the hydrogen and nitrogen are inert, and simply act by excluding the air necessary to the continuance of respiration.

It is difficult to state what proportion of this gas, in a given atmosphere, is required to prove fatal to man; all that we know is that it may destroy life, when in a smaller proportion than one eleventh part. In the accident here reported, ignited bodies continued to burn without leading to explosion; the same has been observed in certain cases which occurred in Paris. Compared with other gases, coal-gas is more powerful in its effects than nitrogen, hydrogen, carbonic acid or carburetted hydrogen; but it is less powerful than carbonic oxide and sulphuretted hydrogen. The post-mortem appearances show that the action of this gas is chiefly confined to the nervous system; the respiratory organs are likewise affected, but more slowly. A peculiar coagulated state of the blood is also met with. This may influence the nature of the symptoms, although it is difficult to say in what way. Can the coagulation be caused by the carbonic oxide or the oily principles? Or may it not be a purely chemical change, produced after death, by the action of these agents? A current of coal-gas was passed through liquid ox-blood without coagulating it. The blood was almost always found coagulated in animals which had been killed by coal-gas, whether with or without its odoriferous principles, and the same appearance was met with in animals killed by carbonic oxide, or carburetted hydrogen. Whatever opinion we may form, it is sufficient to have established, that this compound has a specific influence on the nervous and sanguineous system.

*Annales de Hygiène. Jan.* 1842.

[Remarks. This is a very important contribution to the toxicological history of the gases; and we must compliment M. Tourdes on the careful manner in which the case was investigated. M. Tourdes's experience led him to know the exact points which required to be examined; while it is a very common circumstance that when reports of this kind are drawn up, all kinds of matters are accumulated without reference to what the case is really capable of proving, or in what way it may be made to add to our experience. It is this want of system in drawing up reports, that renders most of those published perfectly useless to the medical jurist; and the profession is therefore the more indebted to the author of the present paper for the care bestowed upon it. There are some points, however, which require a few remarks. It is stated that a mixture of coal-gas with air in a non-explosive proportion (1-15th) will rapidly cause death, and it is inferred that the mixture breathed in these cases could not have been explosive, because there were lighted bodies present. It is also inferred that a mixture in explosive proportions (1-11th of coal-gas) would be irrespirable, and soon destroy life. This inference is scarcely reconcileable with the well-known fact that those employed in coal-mines can work for a length of time by means of a safety-lamp, in spots where a lighted candle would immediately cause an explosion. The gaseous mixture in coal-mines is certainly different in composition from that described by M. Tourdes; but still we do not see that we can fairly connect the explosive properties with the irrespirability of such mixtures. We quite agree with the reporter, that the respiration of mixtures of this kind must be in the end hurtful, even where the gas is considerably diluted; and the cases which he describes probably resulted from the slow action of a very diluted mixture.

The author considers that carbonic oxide is the principal deleterious agent in coal-gas, and perhaps in these cases it was so; but it is necessary to remark, that the Strasburg gas, according to the analysis, contains an unusually large proportion of that compound. It is evident from this analysis, that the gas examined must have been distilled at a very high temperature; and its chemical constitution, therefore changed; for more than one half of it is formed of hydrogen and carbonic oxide. In ordinary gas, the proportion of carbonic oxide fluctuates from 3 to 11 per cent.; and is seldom greater than this, but the gas examined by M. Tourdes, contained nearly double that quantity. Hence these were rather cases of poisoning by carbonic oxide, than by coal-gas; and it is clear, if M. Tourdes's theory were true, we should have to invent some other explanation to account for death, where the coal-gas contains little or no carbonic oxide: for there is no sort of doubt, that in this state, it will prove fatal to life. We look upon coal-gas as a directly poisonous mixture, in which the active agent is liable to vary.

Among the post-mortem appearances, the author of the report alludes to a particular coagulated state of the blood in the heart and great vessels. He found this also in nearly all the animals killed by the gas. We must, however, remark that this is a sign, upon which no reliance can be placed as evidence of poisoning by coal-gas. In two interesting cases of poisoning by this gas, communicated by Mr. Key to the Guy's Hospital Reports, (No. viii, p. 107, April, 1839,) there was *extreme fluidity* of the blood, so much so that *this* was set down as one of the most striking appearances. These two cases occurred under circumstances very similar to those described by M. Tourdes; but the only resemblance in the post-mortem appearances, was great injection of the mucous membrane of the air-passages. These conflicting observations show us, that we have yet much to learn on the subject of poisoning by coal-gas, and that it would be advisable at present to collect facts, without attempting to generalize from them. In relation to medical police, this subject is one of considerable importance: for gas is now introduced into many dwelling-houses, and it is a question which has not yet been sufficiently attended to, namely how far the long-continued respiration of an atmosphere containing but a small proportion of coal-gas, may affect health and life. The evil effects are not immediate; and therefore the respiration of such contaminated air is not considered dangerous or hurtful, but there is no doubt that the health of a person may under these circumstances become insidiously undermined.]

